# Temporal trend of mortality from infectious respiratory diseases in childhood in Minas Gerais, Brazil, 2000-2020

**DOI:** 10.1590/S2237-96222023000300006.EN

**Published:** 2023-10-06

**Authors:** Davi Nilson Aguiar e Moura, Fillipe Reis Silva, David Morosini de Assumpção, Nícolas Emanuel Oliveira Reis, Isabela Fernandes Coelho Cunha, Amanda Priscila de Santana Cabral Silva, Waneska Alexandra Alves

**Affiliations:** 1 Universidade Federal de Juiz de Fora, Departamento de Medicina, Governador Valadares, MG, Brazil; 2 Empresa Pagar.me, Engenharia de Dados, São Paulo, SP, Brazil; 3 Universidade Federal de Pernambuco, Núcleo de Saúde Coletiva, Vitória de Santo Antão, PE, Brazil; 4 Universidade Federal de Juiz de Fora, Departamento de Nutrição, Governador Valadares, MG, Brazil

**Keywords:** Respiratory Tract Diseases, Child Mortality, COVID-19, Time Series Studies, Epidemiology, Enfermedades Respiratorias, Mortalidad infantil, Covid-19, Estudios de Series Temporales, Epidemiología, Doenças Respiratórias, Mortalidade da Criança, Covid-19, Estudos de Séries Temporais, Epidemiologia

## Abstract

**Objective:**

to analyze temporal trends in mortality due to infectious respiratory disease in children under 12 years old in Minas Gerais, Brazil, from 2000 to 2020.

**Methods:**

this was an ecological study using data on infectious respiratory disease in children under 12 years old registered on the Mortality Information System; the variables studied were etiological agent, anatomical site of infection and sex; trends were analyzed by joinpoint regression.

**Results:**

of the 4,688 registered deaths, the etiological agent of the disease was unspecified for 84.5% of them, and 88% were due to lower respiratory tract infections; there was a decreasing trend in deaths and in the proportion of deaths due to unspecified etiologic agents; in 2020, there was an increase in deaths with viral etiology and systemic involvement.

**Conclusion:**

in addition to the change in the etiologic profile, there was a reduction in mortality due to infectious respiratory diseases in children, even considering 2020, the year of the COVID-19 pandemic.

## Study contributions

**Main results:**

From 2000 to 2020, there was a downward trend in mortality due to infectious respiratory disease in children living in Minas Gerais - even in 2020, the year of the COVID-19 pandemic.

**Implications for services:**

There was a reduction in child deaths due to respiratory infections; health services should be alerted as to the considerable presence of ill-defined or inconclusive codes (garbage codes) on death certificates.

**Perspectives:**

It is necessary to maintain the effectiveness of health actions among the mother and child population and to improve the records held on the Mortality Information System, in order to enable better monitoring of mortality as well as to enable analytical studies to be conducted.

## RESUMEN

**Objetivo:**

analizar las tendencias temporales de mortalidad por enfermedades respiratorias infecciosas en niños de hasta 12 años incompletos en Minas Gerais, Brasil, de 2000 a 2020.

**Métodos:**

estudio ecológico con datos de muertes en niños de hasta 12 años incompletos, registradas en el Sistema de Información de Mortalidad; las variables estudiadas fueron: agente etiológico, sitio anatómico de infección y género; las tendencias se analizaron mediante la regresión jointpoint.

**Resultados:**

de las 4.688 muertes registradas, el 84,5% tenía un agente etiológico de la enfermedad no especificado y el 88% se debía a infecciones de las vías respiratorias bajas; hubo una tendencia decreciente en las muertes y en la proporción de muertes por agente no especificado; en 2020, hubo un aumento en el número de muertes de etiología viral y acometimiento sistémico.

**Conclusión:**

además del cambio en el perfil etiológico, hubo una reducción de la mortalidad por enfermedades respiratorias infecciosas en niños, incluso considerando 2020, año de la pandemia de Covid-19.

## RESUMO

**Objetivo:**

analisar as tendências temporais de mortalidade por doença respiratória infecciosa em crianças com até 12 anos incompletos, Minas Gerais, Brasil, de 2000 a 2020.

**Métodos:**

estudo ecológico dos óbitos por doença respiratória infecciosa em crianças com até 12 anos incompletos, registrados no Sistema de Informações sobre Mortalidade; as variáveis estudadas foram o agente etiológico, o sítio anatômico da infecção e o sexo; as tendências foram analisadas por regressão *joinpoint*.

**Resultados:**

dos 4.688 óbitos registrados, 84,5% tiveram agente etiológico não especificado e 88% decorreram de infecções nas vias aéreas inferiores; observou-se tendência decrescente nos óbitos e na proporção de óbitos provocados por agente etiológico não especificado; em 2020, houve incremento do número de óbitos de etiologia viral e de acometimento sistêmico.

**Conclusão:**

além da alteração no perfil etiológico, notou-se redução da mortalidade por doenças respiratórias infecciosas em crianças - mesmo em 2020, ano de pandemia de covid-19.

## INTRODUCTION

Acute respiratory infections (ARIs) are an important cause of morbidity and mortality among children under 5 years of age in developing countries.^1^ Consequences of these diseases include school absenteeism, an increase in the number of medical consultations in the first year of life and poorer quality of life.^2^ Approximately 70,000 children under five years old die on the American continent annually as a result of acute respiratory infections.^3^ On the other hand, Brazil has shown progress and positive results in the area of ​​child health, such as, for example, the 67.6% reduction in the mortality rate among Brazilian children under 5 years old, between 1990 and 2015.^4^ As of 2020, due to the pandemic caused by the novel coronavirus (SARS-CoV-2), a large increase in morbidity and mortality was observed with the emergence of severe acute respiratory syndrome (SARS). In relation to the state of Minas Gerais, the Brazilian Ministry of Health reported an increase of around 650% in the number of deaths due to SARS in 2020, when comparing the data on this indicator to that of 2019, with growth in the proportion of cases of the syndrome associated with the increase in the number of COVID-19 cases in the state of Minas Gerais.^5^

In this context, Death Certificates are of great relevance for the analysis of child mortality, given that they are the main data collection instrument for the Ministry of Health Mortality Information System (*Sistema de Informações sobre Mortalidade* - SIM). Death Certificates must be filled out exclusively by medical professionals, since a qualified clinical and laboratory investigation is essential for defining the underlying cause of death.^6^ However, studies show that a considerable proportion of codes used to record underlying causes of death on the SIM are codes contained in the 10^th^ Revision of the International Statistical Classification of Diseases and Related Health Problems (ICD-10) relating to deaths considered to have ill-defined (Chapter XVIII) or inconclusive causes (garbage codes). Unfortunately this fact makes it difficult to assess the true epidemiological scenario.^6^

This research is justified by the relevance of analyzing the temporal trend of mortality due to infectious respiratory diseases, regardless of the context involving the COVID-19 pandemic. Its results can support decision-making by health managers in directing, planning and distributing public resources. Moreover, as at the time this report was concluded, there was a scarcity of publications using time series analysis of infant mortality due to infectious respiratory diseases in Minas Gerais.^6^

The objective of this study was to analyze the temporal trends of mortality due to infectious respiratory diseases in children under 12 years old, in the state of Minas Gerais, Brazil, between 2000 and 2020.

## METHODS

*Design*

This was an ecological time-series study of mortality caused by infectious respiratory diseases in children under 12 years old, living in the state of Minas Gerais, from January 1, 2000 to December 31, 2020. The study period of two decades was defined with the aim of guaranteeing the quality of the data, since the implementation of ICD-10 by the Pan American Health Organization (PAHO), with effect from 1993, was followed by a gradual process throughout Brazil which differed according to each of the country’s Federative Units.

*Background*

Minas Gerais is the fourth largest Brazilian Federative Unit in terms of territorial area and the second largest with regard to the number of inhabitants. Its territory is composed of 853 municipalities, which is the largest such number among all the country’s Federative Units.^7^ In 2020, 20.8% of the total of Brazil’s inhabitants (211,755,692) were under 14 years old.^8^

*Participants*

According to the Brazilian Child and Adolescent Statute (*Estatuto da Criança e do Adolescente* - ECA), people under 12 years of age are considered to be children,^9^ so that the selection of data used in this study was based on death records held on the SIM referring to the population in this age group living in the state of Minas Gerais. Death records were selected for inclusion in the study that had ICD-10 codes referring to infectious respiratory diseases as the underlying cause of death;^10^ records of such deaths for children outside the selected age group were excluded, as were those for children not living in Minas Gerais and/or whose underlying cause of death was not related to infectious respiratory diseases.

*Variables*

The variables studied were “etiologic agent”, “sex” and “anatomical site of infection”. The latter was used to distinguish infections according to the affected site, that is: lower respiratory tract, upper respiratory tract or systemic involvement. In this stratification, upper respiratory tract infections were considered to be those that affect the initial part of the respiratory tract as far as the larynx, while lower respiratory tract infections are located from the trachea onwards.^11^ Codes that, in addition to involving the respiratory tract, include other organs, were included in the systemic involvement category.

Following initial data collection, the ICD-10 codes were investigated based on clinical grounds and grouped according to the etiological agent causing infectious respiratory disease: 1 - Bacteria (A15-16; A20.2; A21.2; A22.1; A31.0; A38; A42.0; A43.0; A48.1; A78; B90.9; J02.0; J03.0; J13-15; J16.0; J17.0; J20.0-2; J65); 2 - Fungus (B37.1; B38.0-2; B39.0-2; B40.0-2; B41.0; B42.0; B44.0-1; B45.0; B46.0; B59; J17.2); 3 - Virus (B03; B25.0; B33.4; B34.2; B34.9; J00; J09-12; J17.1; J20.3-7; J21.0); e 4 - Parasite (B58.3; B67.1; J17.3). ICD-10 codes corresponding to respiratory infectious diseases that did not clearly determine the causative etiologic agent were placed in the “unspecified” agent category (J39.0-1; J85.0-2; J86.0; J86.9; J01-06; J16.8; J17.8; J18-22; J80; J96.0; U04; U04.9).

In addition, codes related to the anatomical site mainly affected by infections were grouped together as follows: 1 - Lower respiratory tract infections (A15-16; A20.2; A42.0; A43.0; A48.1; A78; A21.2; A22.1; A31.0; B25.0; B33.4; B37.1; B38.0-2; B39.0-2; B40.0-2; B41.0; B42.0; B44.0-1; B45.0; B46.0; B58.3; B59; B67.1; B90.9; J12-18; J20-22; J65; J85.0-2; J86.0; J86.9); 2 - Upper respiratory tract infections (A38; B03; J00-06; J09-11; J39.0-1); and 3 - Systemic infections (B34.2; B34.9; J80; J96.0; U04; U04.9).

The outcomes analyzed regarding the deaths found were the absolute number of deaths (n) and the proportional mortality of deaths, according to etiological agent, sex and site of infection.

*Data source*

The data on deaths, which did not contain the names of the cases, were retrieved from the SIM system on March 31, 2021. They are public data provided by the Ministry of Health, via the Brazilian National Health System Department of Information Technology (*Departamento de Informática do Sistema Único de Saúde* - DATASUS)^12^ ^),(^ ^13^ ^)^ on the following website: https://datasus.saude.gov.br/

*Bias*

In order to minimize possible selection and information biases, the records of the underlying cause of death were selected according ICD-10 codes. Inconclusive codes regarding infectious etiology were discarded. In order to ensure that the deaths related to individuals residing in municipalities in Minas Gerais, we selected municipality codes specific to the state of Minas Gerais.

*Data analysis*

We organized the information using Excel® spreadsheets. Before performing analyses we checked data/variable completeness and consistency. They were included in the study when completeness was greater than 90%.

With regard to proportional mortality, the analysis of data on deaths due to infectious respiratory infections in children took the number of deaths (by etiological agent, by anatomical site and by sex) divided by the total number of deaths and multiplied by 100. Temporal distribution by sex was then carried out.

*Statistical methods*

 The joinpoint regression analysis served to verify trends in the absolute number of deaths and proportional mortality, according to etiologic agent and anatomical site, over the years of occurrence. Joinpoint regression analysis is used to identify the best test model of the various fragments of a line that best explain a trend over time, compared to a single line. The Monte Carlo permutation test is used to define the best model.^14^ ^),(^ ^15^

Using this method, annual percentage change (APC) was estimated, which measures the direction and magnitude of trend results and is used to describe and quantify the trend and assess whether it is statistically significant. APC for each segment is calculated using the following formula,

APC = 100 x (It+1 - It) / I

where I is the indicator in one year (It) and in the following year (It+1). Taking log-scale regression (It) = (b0 + b1t), APC = 100 x (e^b^1 - 1) and calculating the 95% confidence interval (95%CI) using the parametric model,^14^ ^),(^ ^15^ the hypothesis is null if APC = 0, that is, when the rates are stable and their respective confidence intervals are shown.

In our analysis, a maximum of three joinpoints were considered for the periods, so that the percentage change between them allows the temporal trend to be identified as stationary (p-value > 0.05), rising (p-value < 0.05 and positive change coefficient) or falling (p-value < 0.05 and negative change coefficient).^16^

A 5% significance level was used in all the analyses. The statistical analysis was performed using version 4.9.0.1 of the Joinpoint Regression® program, aided by the use of Microsoft Excel® and R 4.1.1.

*Ethical aspects*

The study project was exempt from appraisal by a Research Ethics Committee, since it used only public domain secondary data with no identification of cases. Notwithstanding, the study respected international principles on handling data in research involving human beings.

## RESULTS

A total of 4,688 deaths due to infectious respiratory diseases were recorded for children under 12 years old living in the state of Minas Gerais, during the period studied, resulting in an annual average of 223.2 deaths. Of the total number of deaths, the highest number of records occurred in the year 2000 (n = 527 deaths; 11.2%), considering all the years of the period, while 2020 corresponded to the lowest number of records (n = 78 deaths; 1.7%) (Table 1).

**Table 1:** Time series of number of deaths and proportional mortality due to infectious respiratory diseases in children under 12 years old, by etiologic agent, Minas Gerais, Brazil, 2000-2020

Year of death	Bacterias		Virus		Fungus		Unspecified agent		Total
	No. of deaths	Proportional mortality	No. of deaths	Proportional mortality	No. of deaths	Proportional mortality	No. of deaths	Proportional mortality	No. of deaths
	(n)	(%)	(n)	(%)	(n)	(%)	(n)	(%)	(n)
2000	59	11.2	7	1.3	-	-	461	87.5	527
2001	75	15.7	4	0.8	-	-	398	83.4	477
2002	40	10.9	6	1.6	-	-	322	87.5	368
2003	39	11.4	6	1.8	1	0.3	296	86.5	342
2004	31	10.7	4	1.4	-	-	256	88.0	291
2005	26	10.8	4	1.7	-	-	211	87.6	241
2006	35	12.1	4	1.4	-	-	250	86.5	289
2007	26	12.9	1	0.5	1	0.5	174	86.1	202
2008	21	9.5	8	3.6	1	0.5	191	86.4	221
2009	16	8.2	13	6.6	1	0.5	166	84.7	196
2010	25	14.5	5	2.9	-	-	143	82.7	173
2011	27	17.8	1	0.7	-	-	124	81.6	152
2012	20	11.4	5	2.9	0	-	150	85.7	175
2013	18	11.0	10	6.1	-	-	135	82.8	163
2014	20	15.2	7	5.3	-	-	105	79.5	132
2015	13	10.9	2	1.7	-	-	104	87.4	119
2016	25	16.7	10	6.7	1	0.7	114	76.0	150
2017	12	9.7	3	2.4	0	-	109	87.9	124
2018	21	16.2	9	6.9	1	0.8	99	76.2	130
2019	24	17.4	7	5.1	-	-	107	77.5	138
2020	9	11.5	23	29.5	-	-	46	59.0	78
Total	582	12.4	139	3.0	6	0.1	3,961	84.5	4,688

Source: Mortality Information System/Ministry of Health.

In the period 2000-2020, the highest proportional mortality, according to etiological agent, was attributed to infectious agents classified as unspecified (84.5%). Deaths due to viral agents, which accounted for 5.1% of deaths in 2019, increased to 29.5% in 2020, the year the SARS-CoV-2 pandemic began. The opposite was found in the unspecified agents category, the percentage share of which decreased from 77.5% (2019) to 59.0% (2020) (Table 1).

It is worth mentioning that among the deaths classified in the ICD-10 unspecified etiologic agent category, codes J18.0 (unspecified bronchopneumonia) and J18.9 (unspecified pneumonia) accounted for 76.7% of the total number of records. No deaths resulting from parasite infections were recorded - of these, only six deaths were recorded during the entire 21-year series analyzed.

In the same period, we found that the highest proportion of deaths due to infectious respiratory diseases, in the age group we selected, corresponded to lower respiratory tract infections (LRTI): 88% of total deaths found. Deaths due to upper respiratory tract infections (URTI) and deaths due to systemic involvement accounted for 3.2% and 8.8% of the records, respectively. There was a fall in LRTI and URTI mortality 2020, alongside an increase in deaths due to systemic causes (Table 2).

**Table 2:** Time series of number of deaths and proportional mortality due to infectious respiratory diseases in children under 12 years old, by anatomical site of infection, Minas Gerais, Brazil, 2000-2020

Year of death	URTI^a^		LRTI^b^		Systemic infections		Total
	No. of deaths	Proportional mortality	No. of deaths	Proportional mortality	No. of deaths	Proportional mortality	No. of deaths
	(n)	(%)	(n)	(%)	(n)	(%)	(n)
2000	12	2.3	470	89.2	45	8.5	527
2001	8	1.7	404	84.7	65	13.6	477
2002	10	2.7	314	85.3	44	12.0	368
2003	8	2.3	296	86.5	38	11.1	342
2004	4	1.4	268	92.1	19	6.5	291
2005	4	1.7	210	87.1	27	11.2	241
2006	4	1.4	268	92.7	17	5.9	289
2007	3	1.5	186	92.1	13	6.4	202
2008	9	4.1	195	88.2	17	7.7	221
2009	17	8.7	169	86.2	10	5.1	196
2010	11	6.4	148	85.5	14	8.1	173
2011	4	2.6	137	90.1	11	7.2	152
2012	4	2.3	159	90.9	12	6.9	175
2013	5	3.1	152	93.3	6	3.7	163
2014	4	3	124	93.9	4	3.0	132
2015	8	6.7	106	89.1	5	4.2	119
2016	15	10	127	84.7	8	5.3	150
2017	5	4	111	89.5	8	6.5	124
2018	7	5.4	113	86.9	10	7.7	130
2019	8	5.8	122	88.4	8	5.8	138
2020	2	2.6	46	59.0	30	38.5	78
Total	152	3.2	4,125	88.0	411	8.8	4,688

a) LRTI: Lower respiratory tract infections; b) URTI: Upper respiratory tract infections.

**Figure 1:**
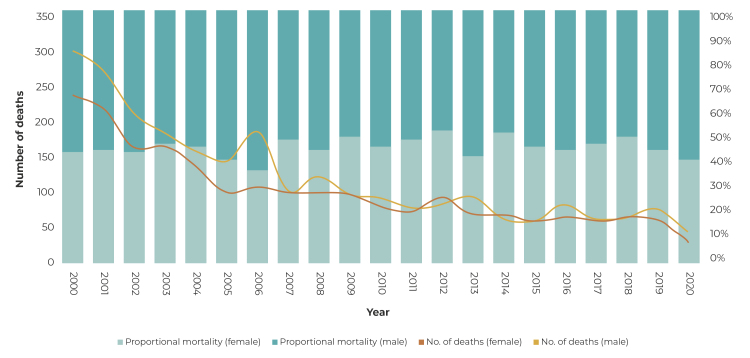
Time series of records of deaths and proportional mortality due to infectious respiratory diseases in children under 12 years old, by sex, Minas Gerais, Brazil, 2000-2020

Source: Mortality Information System/Ministry of Health.

Regarding the sex of the children, proportional mortality due to infectious respiratory diseases showed little variation, with an annual average of 53.9% for males and 46.1% for females (Figure 1). There was a trend towards a reduction in the absolute number of deaths, for both sexes, with a slightly higher number of death records for males (n = 2,551), compared to the number of death records for females (n = 2,131). In 2000, 293 male child deaths and 232 female child deaths were registered, while in 2020 deaths among male (46) and female (32) children showed a proportional reduction of 84.3% and 86.0% respectively (Figure 1).

The temporal trend for frequency of deaths showed a significant reduction (p-value < 0.05) throughout the time series, with negative joinpoints: APC was -13.8% (95%CI -18.4;-8.8) between 2000 and 2005; and -5.7% (95%CI -7.2;-4.1) between 2005 and 2020. As for the proportional mortality profile according to etiologic agent, the trend of deaths due to viral infections showed 11.1% APC (95%CI 5.3;17.3) over the total period, 2000-2020, while deaths due to respiratory diseases attributed to unspecified etiological agents, showed a downward trend, especially after the negative APC joinpoint of -13.0% (95%CI -22.0;-3.0), between 2018 and 2020. Deaths due to respiratory diseases caused by fungal infections were not analyzed, given the absence of records in some years. Regarding the analysis of the anatomical site for deaths caused by respiratory diseases, between 2018 and 2020, we found a tendency towards a reduction in proportional mortality due to lower respiratory tract infections (APC = -16.4; 95%CI -24.6;-7.2) and a proportional rising trend for systemic infections (APC = 155.2%; 95%CI 7.3;507.0), as can be seen in Table 3.

**Table 3:** Temporal trend analysis, using joinpoint regression, of records of death due to infectious respiratory diseases in children under 12 years old living in Minas Gerais, Brazil, by anatomical site of infection and etiologic agent, 2000-2020

Variables		1^st^ trend			p-value	2^nd^ trend			p-value
		Period	APC^a^ (%)	95%CI^b^		Period	APC^a^ (%)	95%CI^b^	
	Deaths (n)	(2000-2005)	-13.8	(-18.4;-8.8)	< 0.001	(2005-2020)	-5.7	(-7.2;-4.1)	< 0.001
Etiologic agent (proportional mortality)	Bacterias	(2000-2020)	1.0	(-0.6;2.6)	0.222	-	-	-	
	Virus	(2000-2020)	11.1	(5.3;17.3)	0.001	-	-	-	
	Unspecified agent	(2000-2018)	-0.4	(-0.7;0.0)	0.029	(2018-2020)	-13.0	(-22.0;-3.0)	0.015
	Fungus^c^								
Anatomical site (proportional mortality)	LRTI^d^	(2000-2018)	0.2	(-0.1;0.6)	0.182	(2018-2020)	-16.4	(-24.6;-7.2)	0.002
	URTI^e^	(2000-2020)	5.8	(1.8;9.9)	0.006	-	-	-	
	Systemic	(2000-2018)	-4.9	(-7.5;-2.2)	0.001	(2018-2020)	155.2	(7.3;507.0)	0.036

a) APC: Annual percentage change; b) 95%CI: 95% confidence interval; c) The data on fungus could not be analyzed due to lack of records for some years; d) LRTI: Lower respiratory tract infections; e) URTI: Upper respiratory tract infections.

Source: Mortality Information System/Ministry of Health.

## DISCUSSION

The findings revealed a falling temporal trend in the number of deaths due to infectious respiratory diseases, a rising trend in proportional mortality due to viral agents, and a rising trend in upper respiratory tract infections among children under 12 years old living in Minas Gerais, according to records for the period from 2000 to 2020.

The literature reveals that Brazil has shown improvement in health indicators in recent decades, especially those related to children,^16^ ^)-(^ ^19^ in particular the study on the trend of neonatal mortality in Brazil which revealed a fall in the period from 2007 to 2017.^18^ ^),(^ ^19^ The results obtained by our study corroborate the evidence on the evolution of health conditions, by pointing to a significant reduction in the frequency of deaths due to infectious respiratory causes in children in Minas Gerais. The importance of the “health conditions” factor can be attributed to the scope of public policy actions at the national level, such as the implementation of the *Rede Cegonha* (Stork Network) and its promotion of a model of health care focused on mothers and children, from prenatal care through to child growth and development. Added to this factor is the very expansion of Primary Health Care and the Family Health Strategy, in addition to the universalization of immunizations.^17^ ^),(^ ^18^ ^),(^ ^20^ Studies have shown that, after the first five years of the implementation of the *Bolsa Família* (Family Grant) Program and the expansion of the Family Health Strategy, with a 10% increase in the coverage of Family Health actions, there was a drop of approximately 4.5% in infant mortality.^21^

From 2019 to 2020 there was a more abrupt reduction in proportional mortality and in the number of deaths due to unspecified agents, and a considerable increase in coronavirus infections (ICD-10 code B34.2). The recording of COVID-19 deaths changed this proportional mortality, with an increase in the viral etiological agent and systemic infection categories, whereby this increased proportion was even more pronounced owing to the reduction in the overall frequency of other respiratory infections.^17^ ^),(^ ^22^ This reduction in the overall frequency of respiratory infections, in contrast to increased coronavirus infection diagnosis (ICD-10 code B34.2), possibly indicates that cases previously classified as being due to an “unspecified” agent began to receive this diagnosis in an epidemiological context of the pandemic.

With regard to etiology, the analysis showed that 12.4% of proportional mortality due to respiratory infection in children under 12 years old of age resulted from infections caused by bacterial agents and 3.0% by viral agents.

 The respiratory tract condition with the highest mortality is the so-called community-acquired pneumonia, which, in Brazil, is more associated with viral than bacterial etiology, due to the good results achieved with the implementation of pneumococcal conjugate vaccines and vaccines against *Haemophilus influenzae*.^23^ ^),(^ ^24^ In this sense, in this study we expected to find more robust values for viral etiology, which proved not to be the case. As the data revealed, 84.5% of the underlying causes of death were reported using “unspecified” agent codes, revealing health service shortcomings in identifying the microorganisms involved in infectious respiratory diseases. This weakness in etiologic diagnosis was also found by an ecological study analyzing infant mortality due to respiratory diseases in Brazil, covering the period from 2009 to 2018, whereby 94.4% of cases had pneumonia due to unspecified microorganisms as the main underlying cause.^19^

With effect from mid-March 2020, actions recommended by Brazilian public health authorities, such as social distancing and isolation, changed the behavior and way of life of families and, consequently, of children. If initially, these measures were aimed at preventing the spread of the infectious respiratory disease in question, i.e. COVID-19, their results may have led to a reduction in morbidity and mortality due to respiratory causes in general.^25^ ^),(^ ^26^ An analysis of the COVID-19 panorama in several countries indicated that morbidity and mortality due to the disease was significantly lower in children than in adults.^17^ ^),(^ ^21^ ^),(^ ^22^ ^),(^ ^25^

As of 2020, our investigation reveals that deaths from respiratory diseases in children continued to decrease, although an increase in the frequency of deaths due to respiratory diseases caused by viral agents was also found, which reinforces national findings published by the Ministry of Health.^27^

Globally, most childhood respiratory infections are self-limiting and are upper respiratory tract infections (URTI).^28^ Lower respiratory tract infections (LRTI), in turn, accounted for around 5% of total deaths worldwide in 2015,^24^ and Brazil accounted for 40% of deaths due to respiratory disease in Latin America, per year.^29^ The findings of our study emphasize this greater relationship between mortality and LRTI.

The records we assessed showed a similar reduction in deaths due to respiratory infections between males and females, also reported in the literature, with only a slight predominance in morbidity and mortality among males due to probable intrinsic (lung immaturity, slow intrauterine development) and extrinsic risk factors.^30^ It has also been noted that male children may be up to 1.5 time more likely to be hospitalized due to respiratory diseases, compared to female children.^28^

There are some limitations inherent to this study that need to be highlighted: firstly, (i) the use of secondary data affected by low completeness and consistency of information recorded on Death Certificate fields, and secondly, (ii) the limitation related to the high percentage of records with underlying cause of death defined using ICD-10 “unspecified” agent codes, which requires caution with regard to interpreting the results.

We therefore conclude that there was a temporal trend towards reduction in the frequency of deaths from infectious respiratory diseases in children under 12 years old living in the state of Minas Gerais, even during the first year of the COVID-19 pandemic, suggesting that public policies focusing on maternal and child health can impact the reduction of deaths from respiratory causes in this age group. However, this profile may differ between the regions of the state, due to the socioeconomic differences between them.

The frequency of deaths recorded in 2020 reinforces, in addition to the natural history of COVID-19 in children, the hypothesis that the regional control and prevention actions adopted during the SARS-CoV-2 pandemic contributed to this trend in proportional mortality due to viral microorganisms and the systemic involvement of the infection.

Furthermore, public investment in the three management levels of the Brazilian National Health System is fundamental, with the objective of improving the records held on the Mortality Information System. This is certainly an important investment for the health care and surveillance sectors for identifying and monitoring mortality among children under 12 years old. Children’s health care, especially in the context of Primary Health Care, should strengthen early diagnosis, timely treatment and, therefore, prevent deaths due to infectious respiratory diseases at this age. More effective health actions are also needed, especially following the COVID-19 pandemic, aimed at reducing factors that influence the chain of events related to child health.
